# Health Emergencies, Falls, and Use of Communication Technologies by Older People with Functional and Social Frailty: Ageing in Place in Deprived Areas of Italy

**DOI:** 10.3390/ijerph192214775

**Published:** 2022-11-10

**Authors:** Maria Gabriella Melchiorre, Barbara D’Amen, Sabrina Quattrini, Giovanni Lamura, Marco Socci

**Affiliations:** Centre for Socio-Economic Research on Ageing, IRCCS INRCA—National Institute of Health and Science on Ageing, 60124 Ancona, Italy

**Keywords:** frail older people, ageing in place, daily living activities, health emergencies, falls, communication technologies, family, neighbors, Italy

## Abstract

Frail older people ageing alone in place need help to perform daily living activities, especially when functional limitations are increasing and formal/informal supports are lacking. This context represents a risk of experiencing health emergencies, in particular falls. It is thus important to understand how seniors manage these potential difficulties and who helps them. The present study aimed to explore these dimensions in Italy, where 120 qualitative interviews were carried out in 2019 within the “Inclusive ageing in place” (IN-AGE) research project, involving frail older people living alone at home. A content analysis was conducted. Results showed that seniors need to manage health emergencies regarding heart and breathing problems but mainly episodes of falls are reported, with consequent fractures and fear of falling again. In several cases, the use of a mobile phone was crucial in order to seek for help, and the first to intervene were children, in addition to some neighbors. Some seniors also referred their ability to call independently the General Practitioner (GP) or the emergency room, in order to not disturb family members. These findings highlight new useful insights for policy makers, regarding health emergencies prevention and management measures to put in place, especially concerning falls, and the support provided by communication technologies.

## 1. Introduction 

The ageing population is often affected by frailty, described as a geriatric syndrome, with increasing physiological decline and functional limitations, especially in performing basic and instrumental Activities of Daily Living (ADLs and IADLs) [1,2]. Overall, frailty is associated with several factors: sociodemographic (e.g., advanced age, low income, living alone, female gender), clinical (e.g., multimorbidity, malnutrition, impaired cognition), and lifestyle (e.g., physical inactivity) [3,4,5]. Frail older adults are thus vulnerable to several illnesses and disabilities [6,7] and are at higher risk of reporting dependency, hospitalizations and institutionalization [8,9,10]. When frail older people with limited informal and formal care supports are ageing alone in place who are looking for maintaining their autonomy in their place of residence as well as to participate in their community, even though living without cohabitant family members, it the management of possible health emergencies becomes crucial, such as serious medical illness (e.g., heart/circulatory problems), physical complaints and falls. Several difficulties in particular occur for older people who manage their health at home despite the increasing age and multiple chronic diseases, which can lead also to immobility over time [11]. Emergency situations overall put older persons at risk, since poor health conditions, possible social and economic weakness, and scarce care support, can hamper their ability to react in this respect [12]. In particular, regarding falling, biological (e.g., age, co-morbidity), behavioral (e.g., sedentary behavior), environmental (e.g., hazardous features at home and in public environment), and socioeconomic (lack of social interaction, limited access to health and social services) risk factors are indicated [13]. Moreover, the risk of a fall, due to mobility issues and poor balance, is higher for seniors with arthritis, malnutrition and loss of muscle strength reducing physical/mental functions; other factors are obesity, decreased visual acuity, depression, cognitive impairment/absence of orientation, and tremors due to Parkinson’s disease [14].

Worldwide, mainly older people report falls [15]. In particular, approximately 30% of seniors aged 65 years and older and 42% of those of 75 years and over fall at least once a year. The relationship between frailty and falls represents a crucial challenge for ageing [16], due to the existence of a “vicious cycle of functional decline with frailty leading to falls, greater frailty and more falls” [17] (p. 2242). Kelsey and colleagues [18] reported that approximately 54% of falls occurred indoors and 47% outdoors. Moreover, 77% of indoor falls occurred in seniors’ own home, whereas outdoor falls mostly occurred on sidewalks (23%), gardens (14%), and streets (14%) [14]. Falls result in hip/bone fractures, traumatic brain injuries, damages to intra-thoracic and intra-abdominal organs, joint distortions and dislocations [14]. Often falling implies the need of visits in emergency department, also leading to higher mortality among seniors aged 65 years and over [19,20]. According to Bauer and Kisser [21], in Europe approximately 60% of non-fatal injuries of seniors occur at home, where more than 70% of all injuries are falls, the latter representing the major cause of unintentional deaths for older people.

Falls may even lead to a post-fall syndrome, including confusion and depression [13], in addition to the fear of falling, with strong impact on the quality of life of seniors [22]. Fear of falling derives from difficulties to get up after a fall, suffering from fractures, being hospitalized, and shame [13]. Fear of falling can lead to loss of confidence and self-esteem, to self-impose some restrictions of physical and social daily activities, with further decline of physical capacities, increased risk of falls and possible occurrence of future episodes [3,23]. Furthermore, falls in older age significantly impact healthcare and related costs all over the world [13]. After a fall, seniors experience indeed long hospitalizations, complex treatments, thus needing resource-intensive care, including rehabilitation and home care [24,25], in addition to effective discharge planning interventions [26].

In Italy, the country where this study is focused on, the proportion of older people aged over 65 years is approximately 24% of the total population, as of 1 January 2022 [27]. These seniors represent half of citizens living alone, and 44% of them present severe functional limitations [28]. In this country, over a third (36%) of domestic accidents concern a person aged 65 and over, and among older sufferers the incidence of falls is 77% [29]. Some research findings report that, in the four-year period 2017–2020, in Italy, 8% of seniors aged 65 years and over fell in the 30 days prior to the interview, with 18% of them being hospitalized for at least 1 day. Furthermore, 24% of them referred depressive symptoms, and 39% were fearful of falling. Only 16% received advice from a medical doctor or a health care worker on how to avoid falls [30]. Previous data [31] report that the number of Italian oldest patients aged over 80 years, who get access to emergency rooms following accidents/falls, increased of 60% in 10 years (2005–2015). In Italy, seniors often fall into the house, especially in the kitchen (25%), in the bedroom (22%), on stairs (20%) and in the bathroom (13%). In fact, the main causes of falls are related to the environment (31%), in addition to gait disturbance/reduction of muscle strength (17%) and dizziness/vertigo (13%) [32]. In this country frail older people receive overall support (not specifically for emergencies) mainly from their children and other relatives (78%), whereas 42% report help from private services, 42% from friends/neighbors, 36% from public services, e.g., home care (*Servizio di Assistenza Domiciliare*—SAD), and 23% from personal care assistants (PCAs) [33], with some respondents reporting more than one type of support. Only 2% of seniors live in residential care facilities [34].

Overall, older people need to feel safe in order to live independently longer in their homes. In this respect, communication technology represents a good support for ageing in place [35], and it can be useful for preventing/reducing the consequences of adverse events for health, such as falls [36]. In particular, remote health monitoring, fall alert detectors, and various alarms, can improve older people’s safety at home [37,38].

In order to explore health emergencies, especially falls and the use of communication technologies in this regard, focusing on frail older people ageing in place in Italy, the paper aimed to answer the following research questions: (1) Which main health emergencies (e.g., physical complaints/illnesses, falls) seniors experienced in the last 3 years? (2) Do they use communication technology in general and for facing emergencies (e.g., mobile, smartphone, alarms)? (3) Who provided the first help in emergency circumstances (e.g., family members, friends/neighbors, PCA, or nobody)? To our knowledge, our study on health emergencies, falls and use of communication technologies of frail older people ageing in place alone, is novel, and thus provides a contribution to the related research field. The analysis of these aspects can be indeed of help in order to understand main difficulties faced by seniors in such circumstances, especially when help was not available/requested, including the possible support provided by the use of technological instruments for seeking help. These findings can highlight main risks in this respect, with new useful insights for policy makers, in particular for fall prevention/management, and overall health promotion of older people, by encouraging and concretely supporting their ageing in place.

## 2. Materials and Methods 

### 2.1. Study Design, Participants and Recruitment

The results of this study come from the “Inclusive Ageing in Place” (IN-AGE) qualitative research project, aimed at involving 120 Italian older people (both men and women) aged 65 years and over. Participants were recruited in three Italian regions: Lombardy in the North, Marche in the Centre, and Calabria in the South. These regions are representative, respectively, of high, medium and low levels of socio-economic development of the country [39,40]. The inclusion of these regions thus provides an overall picture of three main contexts in this respect in Italy. In each region, one medium-sized urban city (100,000–200,000 residents, i.e., respectively, Brescia, Ancona and Reggio Calabria) [27], and one inner/rural area (i.e., respectively, Oltrepò Pavese, Appennino Basso Pesarese Anconetano and Area Grecanica) were selected. In particular, inner areas were defined by both the national *Agency for Territorial Cohesion* [41] and the *National Strategy for Inner Areas* (NSIA) [42], as peripheral and disadvantaged rural sites that are not easily accessible (according to their geographical distance from the most served urban poles). Within both urban and rural contexts, the most fragile locations were considered. These were defined by some characteristics, as follows: greater presence of older people living alone; greater presence of families living in public housing (*Edilizia Residenziale Pubblica*—ERP); high unemployment level; low education level; and low availability of public services, especially for older people [43]. Since a holistic approach to frailty, as described in the Introduction, was complex to manage, our study adopted a non-exhaustive/limited definition, leading to a more accessible analysis of this dimension [33], referring to an overall condition linked to ageing, limited abilities in daily living activities (physical/functional frailty), and lacking/scarce support in this respect (social frailty), especially when living alone [44]. Thus, no standard tool was used to assess frailty, and this dimension was ‘asked’ instead of measured. Inclusion criteria for participants were the following: older people living at home alone or with the support of a PCA; intermediate mobility within the home and outside with help (from a person or aids); no cognitive impairment, in order to answer interview questions independently; and absence of very close family members (living in the same urban block/rural building) giving support for daily activities. According to these criteria, a purposive sample was built [45], with the aim to find 72 participants from urban cities (24 each), and 48 from rural sites (16 each), for a sub-total of 40 in each region. We built such a sample with subjects selected in order to assure a typological (not statistical) representativeness. Thus, 40 participants in each region, and a total of 120 (24 urban and 16 rural, in order to respect the lower population numerosity in internal areas) were considered suitable and sufficient in this respect. This number of participants was mainly due to the fact that this was a qualitative study aimed to deepen the individual experiences by collecting and recording narratives, as designed in a semi-structured topic guide, and not a quantitative survey, usually allowing larger samples requiring statistical representativeness. Local sections of voluntary associations (e.g., Auser, Anteas, Caritas), and operators of SAD, were of help for recruiting respondents. The territorial contacts of the recruiting channels built a preliminary list of potential eligible seniors meeting the inclusion criteria, and subsequently, first by telephone and then personally, verified their availability, to be involved in the research, on the basis of a detailed information letter on the purpose and method of the study, data use, and privacy/anonymity of the information collected. These contacts also required preliminary verbal consents of seniors to be interviewed, and also to communicate their address and telephone numbers to interviewers. These operators/providers verified in particular the presence/absence of cognitive capacity and intermediate mobility of participants, in addition to very close family members who give help, by carrying out initial screening/pre-interview based on their own information and assessment, and on their periodic access to the seniors’ home. These aspects were then confirmed by the interviewers (psychologists and sociologists), and further checked also with respective relatives of the older persons interviewed. However, of the 208 involved seniors, 88 (42%) declined because, when contacted to set a date for the interview, they were ill, or were not available during the data collection period for other reasons, or did not feel fully comfortable having an interview and discussing some private issues, even though a preliminary consensus was given. Moreover, while we decided to have a sample aged 65 years and over, in the final sample any respondent aged 65–66 years was included. Thus, the sample of participants started effectively/de facto from 67 years (as ‘youngest’ seniors).

### 2.2. Ethical Considerations, Data Collection, and Measures

Before starting the study, the approval of the Ethics Committee of Polytechnic of Milan was obtained (POLIMI, Research Service, Educational Innovation Support Services Area, authorization n. 5/2019, 14 March 2019), and a written informed consent was signed by each senior. Participants were carefully informed on privacy and anonymity of the information collected, according to ethical issues indicated by the European General Data Protection Regulation (GDPR) n. 679, of 27 April 2016 [46]. In May–December 2019, qualitative face-to-face interviews were administered by six researchers (mainly psychologists and sociologists), by using a semi-structured interview, with questions (both open-ended and closed) on socio-demographic aspects, health status and functional limitations, health emergencies and use of communication technology, care arrangements and use of services, housing and economic situation. The topic guide was built with questions also drawn from previous similar studies e.g., [47], mainly with regard to general aspects, such as socio-demographic, health status and functional limitations, care arrangements and use of services, and validated tools to assess functional limitations, i.e., ADLs and IADLs scales [48]. Questions on difficulty in seeing, hearing, going up/down the stairs, and bending to pick up an object [49,50] were added. As for health emergencies, the questions were the following: (1) Thinking about the last 3 years, did you experience an emergency situation with health consequences? (e.g., fractures, respiratory crisis, flu, heart attack, stroke)? (2) Did you ask for/need help from someone? (3) Thinking about the use of modern tools and technologies (e.g., mobile/smartphone, alarms), can you tell me if you use them in general and in case of emergency? The choice to set a time limit to the 3 years preceding the interview, is due to the need not to consider only the present moment, in order to collect retrospective data, since some health emergencies can have consequences over time, but also to the need not to go too far back in the years and risk respondents not remembering enough of related episodes. Previous falls are however important predictors of new/future falls [51].

### 2.3. Data Analysis

Interviewers audio-recorded and transcribed verbatim all the narratives. Appropriate codes were used to replace name, address and telephone number of respondents [33]. Moreover, the following steps of the Framework Analysis Technique [52,53] were followed: reading the transcribed narratives; identification of macro-sub categories/themes; indexing-labelling; construction of a thematic chart; interpretation of contents. A thematic content analysis [54] was carried out manually, by means of thematic charts (a two-way matrix) where narratives were broken down (rows corresponded to cases/interviewees and columns corresponded to categories), without using software, as also allowed by literature, in order to become more familiar with the results [55,56]. This phase was supported by a topic guide whose questions acted as preliminary conceptual framework, since they were based on both the literature and experience of researchers involved in the research project [33,57], and were used as theoretical-based categories relevant to the topic of ageing in place explored in the study. For the overall analysis, the cell color-coded process allowed grouping data, according with the color assigned to each category [58]. In total, 6 members from the research team filled in the charts, and analyzed results of all 120 narratives. All researchers of the project’s consortium discussed the adequacy of coding for the contents [33]. Statements were counted and presented in tables, for resuming results. However, some absolute values, regarding details/statements which were referred by few participants, are reported in the Section 3 as simple frequency of labels in selected answers, without a reference table [33] (e.g., remote alarm/call devices not owned/wanted, not owned/not wanted; copy of the home keys to family/friends/neighbors; “do not disturb/worry” the family, “family cannot be of help” as reasons for not seeking help from the family). For the analysis, the following main categories/labels were examined (Table 1). 

With regard to the use of communication technologies for seeking help in case of health emergency, and people who gave help in such circumstances, a distinction between various illnesses and falls (and regarding the latter, at home and outside the home) was not elaborated, since respondents often reported more episodes, but indicated the overall use of devices and support received. Furthermore, friends and neighbors were considered together because the two categories often coincide, especially in old age, as reported by the interviewees themselves.

The overall analysis was integrated by original statements/quotations, which emerged in the transcription of the interviews, and coded with IT (for Italy) and progressive interview number (1–120). In order to facilitate the understanding of the quotations, a small amount of editing was done, without however altering the meaning. In particular, empty round brackets indicate not relevant omissions, and square brackets include some words which were added for adjusting the sentence construction. Further details on the Methods (setting, sampling, participants, data collection, measures, and data analysis), can be found in a previous own publication of authors (Melchiorre and colleagues, 2021), with the same study population [33] from which the Section 2 has been partly adapted.

## 3. Results

### 3.1. Main Sample Characteristics

The sample includes 120 respondents, whose main collected characteristics are the following: 80 years and over, women, low/medium level of education, widowed, living alone without cohabitant relatives or PCA, mild/moderate level of physical limitations, mobility also outside the home, and greater support from not cohabiting family members, especially children (Table 2). More detailed information on the sample is available in a previous own publication of authors (Melchiorre and colleagues, 2021), with the same study population [33].

### 3.2. Health Emergencies in the Last Three Years: A Quantitative Map of Statements

#### 3.2.1. Falls and Physical Complaints/Illnesses

In 50 cases various physical complaints/illnesses, as emergencies to be managed, were referred, mainly due to the following reasons: heart/circulatory problems and heart attacks; breathing problems, cough, bronchitis; strong joint problems; stroke/ischemia; and few cases of other complaints (flu, tonsillitis, fainting, inflammation, anxiety). Moreover, 47 respondents reported at least one fall in the past 3 years, mainly at home. Thirty-two older people do not report/mention any health emergency in general (including falls), or they refer mainly health problems resolved over time with medical/specialist cure, or with hospital admissions/scheduled surgery, however not defined as emergencies (Table 3).

Falls in particular often caused fractures (28 cases), and generated some fears, such as falling in general/again (18 cases), also due to broken sidewalks and streets, which make going out difficult, with a greater risk of falling. More consequences of falling were reported by some seniors. There is also a need for walking aids and to hire a “monitoring” PCA in some cases, as a consequence of previous falls (Table 4).

#### 3.2.2. Using Communication Technologies in General and to Ask for Help

Overall, 19 seniors use independently only a landline telephone, 80 the mobile phone, and 21 the smartphone. Only 13 respondents report to own and use remote alarms/call devices (Table 5).

These communication tools, in particular the mobile, were essential to call for help in case of health emergencies, especially if they occurred at home while seniors were alone, as reported by 40 of them. Only in three cases the use of remote alarms (including a mobile with SOS option) was referred with regard to an emergency, whereas 10 seniors possess these devices but do not specify the related availability when the emergencies were experienced. There are also five older people who do not own but would use a remote alarm and conversely two reject it at all (data not shown in the table).

#### 3.2.3. First Help for Health Emergencies

As indicated in Table 6, the first help (asked and received) comes mainly from the family (52 cases) and then from the overall community.

In fact, in most of the emergency situations reported, mainly children (38, both male and female, when specified) were of help, in addition to nine nieces/nephews (most often children of sisters/brothers) and five sisters/brothers. Friends/neighbors (21 cases) and PCAs in four cases also supported the seniors. Other figures were further helpful, such as eight passersby/unknown people in case of falls outside the home/on the street. These in turn called the GP, the medical guard or the emergency room when necessary, and warned family members too. It also emerges in three cases (data not shown in the table) the importance of having provided relatives and friends/neighbors with a copy of one’s home key. This “strategy” indeed allowed seniors to be rescued. In 17 cases the overall ability to deal independently with an emergency is reported, e.g., to seek promptly for public health help (e.g., by contacting GP, emergency room) by themselves (11 cases). In six cases even no help was sought, in order not to disturb/worry the family (three cases) and also because relatives/children cannot be of help, especially if they work (three cases) (data not shown in the table).

### 3.3. Health Emergencies in the Last Three Years: Quotations from the Narratives

#### 3.3.1. Physical Complaints/Illnesses

Main health emergencies are due to heart/circulatory problems and heart attacks, that often required the use of the emergency room, or the intervention of cardiologists directly in the seniors’ home.

*I had a heart attack (…), and my family called the cardiologist, who came here at home. (IT_104)*

*One year ago I had an episode of arrhythmia. On this occasion, the GP measured my blood pressure and called the ambulance to go immediately to the hospital. (IT_86)*

Episodes of cough and bronchitis also emerged. These required a quick intervention with consequent hospitalization, especially in the presence of severe breathing/respiratory crisis.

*It happened three years ago. I was sleeping and I felt that I was breathing not so well. I thought it was flu but when I could no longer breathe [due to a bronchitis], my children put me to the hospital, where I was treated for 10–12 days. (IT_98)*

*The last episode happened on Christmas (…). I had a persistent cough! I felt very bad (…). I had a short breathing, so I went to the hospital and the doctors diagnosed me with asthmatic bronchitis. (IT_37)*

In addition, sudden joint problems can cause emergency health situations, and events such as stroke/ischemia have produced still visible signs and consequences.

*I had severe pains on the back! As if someone was putting a knife inside (…). My family called immediately a physician. (IT_64)*

*My lips deformed when I had a stroke. Since that time, I have not been able to walk alone any more! (IT_119)*

An episode of adverse drug reaction also emerged, due to incompatibility between some pharmacological treatments prescribed by the GP.

*I called the GP because a drug was making me sick. I was feeling dizzy, I couldn’t stand up. I was also depressed for the situation! (IT_112)*

#### 3.3.2. Falls in the Home and Outside

As for falls at home, some older people remained on the ground for many hours before being rescued, mainly by family members and neighbors.

*I realized that suddenly my legs crossed (…). I clung to the door but I went down [to the floor]. I couldn’t get up any more, I spent four hours on the floor, before calling a neighbor. (IT_53)*

*I fell to the ground, I spent two hours on the ground because there was no one who could help me. I was screaming and no one could hear me! Then a friend [who is also a neighbor] came! (IT_115)*

As for falls outside the home (with help from passersby/unknown people), the main cause is represented by broken sidewalks and streets.

*Sometimes there are parts of the road that are a bit sloping, full of holes, and persons cannot walk well. Once I fell quite there! (IT_64)*

*Two years ago I fell on the street because there was a hole. I bent down to pick up something, I skidded and slipped to the ground. (IT_59)*

Sometimes seniors risk falling because they are hit by a car.

*Three years ago (…) a car hit me when I was walking on the pedestrian crossing. I went on the ground with pain in the pelvis. Now I cannot breathe well, and I cannot do the climbs! (IT_86)*

#### 3.3.3. Consequences of Falls

##### Fractures

Following falls, sometimes however without specifying whether at home or outside, joints fractures are often reported, in particular of the femur, shoulder, and limbs in general.

*I slipped into the house, tripping over the carpet, and thus I broke my shoulder. (IT_35)*

*Last year I was going down the stairs and suddenly my hands slipped and I fell. Due to this fall, I had a surgery on my foot. (IT_115)*

*I often fell and broke my knee, ribs and femur! (IT_83)*

Sometimes a fall does not cause fractures but can break dentures, which are fundamental to many seniors.

*Once I fell to the floor in the kitchen and broke my dentures. I had to make it new. I went through three months of hell! (IT_118)*

##### Need of Aids and PCA

After a fall, especially when this is leading to fractures, it is often necessary to use aids for mobility/walking, or to hire a PCA for “monitoring” and support.

*I am in a wheelchair because I fell and broke a femur. Some time later I fell again and broke the other femur! (IT_60)*

*I use a cane for walking after two falls and two fractures. (IT_52)*

*I fell and broke my femur (…). I still did not have a PCA, but after this episode my son said I could not live alone anymore. (IT_119)*

##### Fear of Falling

As a result of falls and fractures, older people are also strongly afraid of falling again, both at home and outside, with a consequent additional fear of moving alone, especially outside.

*Whenever I take a shower I am always afraid of falling! The first time I did it [alone] I fell! (IT_36)*

*Until a couple of years ago I used to go out every day. After a fall, I do not feel like going out alone anymore, I am afraid of falling! (IT_58)*

A senior is particularly worried about falling, because in this case his children have to take care of him, and he does not want to disturb them too much.

*I am sorry if I fall, thus I am very careful, especially at night, since I think “If I fall, I need help from my children!” (…). It would be a nuisance to call them for this! (IT_59)*

##### Fear of Broken Sidewalks and Streets

Older people refer fall outside the home also due to inadequate maintenance of both sidewalks and streets. This aspect generates a lot of fear for their own safety. In particular, there are fears of holes and cobblestones, which make walking dangerous and further increase the risk of falling.

*After I fall I am terrified of holes in the streets. (IT_52)*

*The sidewalks are broken, the road is full of holes. I have to be careful and look very well where I put my feet, since I risk falling! (IT_92)*

*The cobblestone flooring is always dangerous, it is not always well maintained, and this increases the risk of falling. (IT_79)*

The fear of going out and falling also implies the extreme fear of not being able to walk anymore. However, comfort and encouragement, to try anyway (to walk), may derive from living perhaps with a spouse. This does not seem longer possible for those who are widowed.

*If one starts walking again [after falling], but he is afraid to go out, he will not walk anymore (…). When one become older, infirmities are many (…). However, when one lives still in company [with a wife] it is different, there is more security and reciprocal help! (IT_51)*

### 3.4. Opportunities from Communication Technology

#### 3.4.1. General Use

Seniors use mainly mobile phones to communicate, and less often smartphones. However, both are used almost exclusively to call children and grandchildren, whose numbers have been stored in the first positions of the phone book.

*I use my mobile phone to call my children and grandchildren. I use it only for this, for the family. I always wear it attached to my neck [by means of a phone holder]. (IT_109)*

*I have memorized all the numbers of my children and grandchildren in the smartphone. (IT_118)*

*I communicate with my children by means of WhatsApp, I read the messages and see the pictures they send. (IT_98)*

However, some seniors do not know how to use the mobile phone or the smartphone, while others even consider them harmful to health.

*I cannot use the mobile, I am not able to dial the numbers! (IT_116)*

*I have a smartphone, but I do not like it. I am afraid of it, because in my opinion it is dangerous for my health! (IT_111)*

Less respondents report the use of remote alarms/devices.

*I have this [device]. If I fall down I push the doorbell that is connected to my childrens’ phone. (IT_10)*

*To call my daughter, I use my mobile phone with the SOS button. If I press it, immediately her [registered] number is called. I always keep it with me. (IT_110)*

#### 3.4.2. To Ask for Help in Case of Emergencies

The actual use of communication technology, even in case of health emergencies, overall focuses on the mobile phone, which has often been essential to call for help, despite vision problems in some cases.

*Then [after the fall] I reached out my mobile phone and called my daughter. (IT_99)*

*I felt sick during the day and I called my children by mobile. I have all the numbers registered in the phone book but I have difficulty finding them because I cannot see very well! (IT_100)*

Conversely, when seniors were not able to reach and use the mobile phone after a fall to ask for help, their rescue was delayed.

*Immediately after the fall I could not call anyone because I could not reach my mobile, and moreover I had not my glasses! (IT_53)*

*When I fell to the floor, at the moment I did not call anyone for help, because I did not know where I had my mobile phone! (IT_52)*

Remote alarms, even though they are possessed by 13 respondents, were used only in three cases of health emergencies.

*Once I was practically no longer breathing [due to a bout of bronchitis]. I was able to get up and sound the alarm. My daughters came, and then the ambulance arrived in five minutes and they (ambulance personnel) gave me oxygen. (IT_20)*

In five cases, these remote devices are not owned but “wanted”. Seniors seem convinced of their usefulness, but also fear their possible uncomfortable wearing, in addition to a lack of sufficient information for the purchase.

*It would be useful to have an alarm. However, then I should always keep it around my neck! (IT_43)*

*I heard that there is a kind of bell that rings in case of need and I would like to have it (...). I have to inform myself well, I do not know who to contact to buy it. (IT_87)*

Two ladies report differently that these devices are not owned and “not wanted”. They think that living in a nursing home is better than living alone with a remote alarm, when seniors are no longer independent.

*I have never had such an alarm, because I do not feel the need (…). As long as I can, I manage my health by myself. When I realize that I am no longer able to be independent, I go to the nursing home! (IT_29)*

### 3.5. First Help for Health Emergencies

#### 3.5.1. Family: Children, Niece/Nephew, Sisters/Brothers

As already emerged from some of the statements reported above, children are the first to be called and intervene for helping, if possible. The children then arrange to call the GP or the public emergency service, if necessary.

*I got sick here at home and I called my daughter on the mobile phone. She came here immediately and called an ambulance. (IT_117)*

*One morning I just could not breathe anymore (…). However, I was still holding on with my brain, and thus I phoned my daughter and told her to come because I felt bad! (IT_98)*

It should be emphasized that sometimes seniors hesitate a little before “bothering” their children in case of emergencies, especially if they work or do not live nearby.

*When I fell I could not get up and slept overnight on the ground so as not to disturb my daughter. I called her in the morning because then she has to go to work [and she has to sleep at night]. (IT_99)*

Even nieces/nephews and brothers/sisters intervened at times.

*I felt bad at home [due to a stroke]. On this occasion my nephew helped me. He came and took me to the hospital. (IT_85)*

*When I needed for health emergencies, my sister and my brother did a lot for me! (IT_15)*

#### 3.5.2. Friends/Neighbors and PCA

Some of the statements reported above have also anticipated that neighbors/friends have been fundamental in some emergency situation, especially in case of falls and when older people have been able to use the telephone to call them for help.

*When I fell I could not get up. I have a neighbor/friend who works in the emergency room. I called her on the phone, she helped me, and she took me to the hospital. (IT_33)*

*Once I fell, I called on the phone the neighbor who lives downstairs, and she came up fastly! I crawled on the ground and opened the door. (IT_36)*

In particular, neighbors, and in some cases the PCA when present, were important in the immediate rescue, and then proceeded to warn family members or the GP after the incident.

*I fell and called a neighbor who came to help me. Then she called my children. (IT_119)*

*I fell home twice but the PCA was with me and called the GP. She was precious for me! (IT_103)*

#### 3.5.3. Further Aspects Regarding the Help Received

In three cases, having previously given a copy of the home keys to family and friends/neighbors, proved to be a fundamental choice to be rescued.

*[After the fall] I slowly got my glasses and called my sister by mobile phone. She has the keys of my house. (IT_53)*

*When I fell I called a friend of mine who had the keys to the house and asked her to come and pick me up. (IT_95)*

Moreover, as for falls outside the home/on the street, passersby/unknown people intervened on the first moment, and then relatives were warned.

*I fell down in the street and a passerby helped me up and called my daughter (IT_59)*

#### 3.5.4. “Do-It-Yourself”

In case of need, “do it yourself” also emerges, with autonomous management of emergencies. In these cases, the interviewees called for help by themselves, without asking support to family members or neighbors.

*I got out of bed and hit my head against the wall. I did not have anything so serious, but for fear I called the emergency room by myself. (IT_25)*

Public health aid is called by seniors themselves, in particular when they believe that family/children, or other relatives, cannot be of immediate help in case of need, and moreover they do not want to disturb them, especially if they live far away.

*I felt sick at home and called the emergency medical service. I am alone at night. There is no one, this is a big problem (…). My son, even if I call him, cannot come. He works, then he has his wife and a daughter. He cannot neglect them to assist me! (IT_88)*

*I called the Red Cross by myself (…). Even if I call them [children] I just scare them. They live far away! (IT_14)*

Some interviewees seem however resigned to their condition as older people living alone, and thus they did not seek for any type of help when health emergencies were experienced.

*I fell on the balcony and then slowly I got up by myself (…). It is not the first time. This is my life, I live alone! (IT_83)*

Two seniors are conversely worried about a possible “do it yourself” in case of emergency, especially at night, because nobody could be of help.

*If I feel bad at night I do not know who could help me! This worries me very much! (IT_96)*

## 4. Discussion

The aim of this study was to explore health emergencies experienced by frail older people ageing in place in Italy, especially falls and the use of communication technology for seeking help. As people age they become indeed frail, and present several functional/physical limitations with difficulties in performing the basic and instrumental activities of daily living (functional frailty). These limitations hardly impact their capacity to react to health emergencies, especially when seniors live alone and do not have available support from family members, friends and neighbors, as well as from services (social frailty). Despite our study used such a simplified definition of frailty [33], how frail seniors often experience various physical complaints/illnesses and mainly falls, with consequent fractures and needs of aids, emerged from our overall results. Children are main supporter in these circumstances, but sometimes older people prefer to call independently the GP or the emergency room, in order to not disturb the family. These different approaches, and main difficulties and fears of seniors, are deeply discussed below. It is to premise that specific data on older people living alone, and facing health emergencies in Italy, are not always available in the literature. Therefore, also data on general aspects of seniors aged over 65 years and regarding Europe (e.g., falling issue), similar to those reported in the Introduction, have been considered in order to discuss the findings.

### 4.1. Health Emergencies: Falls and Other Complaints/Illnesses

Respondents who experienced emergencies in the past 3 years, related to sudden physical complaints/illnesses, mainly reported circulatory/heart and breathing/bronchitis problems, but several also referred at least one fall. Accordingly, other authors [59] indicate that older individuals are more likely to experience falls and various health emergencies, e.g., increasing blood pressure and decreasing respiratory capacity, these representing the most common causes of death among older people [60].

From our results, the need of services from emergency room or GP in general, due to health emergencies, also emerged, as confirmed by literature. It is indeed known that seniors represent a significant percentage of users, with conspicuous consumption of resources due to the high use of radiological and laboratory diagnostic tests [61], often following crises related to known diseases or serious events [62]. Results from a study in Finland [63], involving approximately 6944 patients aged 80 years and over (median age 85 years), representing 1.5% of the local population, showed that these seniors made 17,769 emergency department visits during 2 years before the survey, accounting for 15% of all visits (118,076) in the department itself. Authors state that these results show a “high incidence of emergency department visits in older patients” [63] (p. 4). A systematic review highlighted in particular that the geriatric population, when assuming a high number of prescribed drugs and with heart disease, frequently use emergency-department services [64]. Further literature [65,66] states that 25% of seniors aged 70–79 years suffer from adverse drug events, vs. 4% among those younger aged 20–29 years. In our study only a person reported an episode of adverse drug reaction, due to incompatibility between some pharmacological treatments prescribed by the GP. However, this highlights the presence of a serious and general issue in this respect. It could be overall assumed that the population, especially seniors, are not aware that some of their health problems could depend on drug interactions, and thus they do not report possible related episodes as such.

In addition to acute and exacerbated pathologies, our respondents reported several episodes of falls at home. Many authors highlight that falls are “tragic events”, a typical and complex “geriatric pathology” generating functional decline and hospitalization/institutionalization. Falls are among the main causes of death in the elderly [30], and trauma from falling, besides mental/cognitive pathologies, are among the main reasons leading Italian older people to emergency rooms [67]. Some studies refer that falls represent the most frequent and serious accident/injury especially for seniors aged 65 and over [68,69], that is the overall starting age-range for selecting of our sample. Moreover, similarly to our findings, data from Epicentro [30] reveal that 63% of episodes of falls of seniors occur at home, and only 21% outside/on the street. Further literature puts in evidence that older people fall at home mainly when entering/exiting, walking on irregular pavements, climbing stairs and using the bathroom [70,71]. These data in turn call into question the crucial issue regarding home safety. In this respect, some authors [72] stress the concept of “lack of perception” of seniors regarding existing architectural barriers in their homes, which can be responsible of falls, even though in Italy 76% of older people live in buildings with barriers. This could depend on a consolidated habit and adaptation over the years to the characteristics of their living space, both indoor and outdoor. Regarding the latter, seniors in our study fall (even though less frequently) in public spaces and on the road due to broken sidewalks and streets. Accordingly, some authors indicate environment being threatening/unsuitable and poorly accessible for seniors, when there is no/scarce maintenance, in cities that are too often hostile and insecure places [72,73]. Other authors [6] found that outdoor falls accounted for approximately 50% of falls among older people aged 80 years and older, and occurred most often due to environmental hazards, and when seniors were walking (47%). In this respect, functional training and educational materials from health professionals/geriatricians, on barriers for walking, seem positively impact the prevention of falls among seniors, thus helping them to maintain balance [14]. Comprehensive balance/mobility training activities, in order to manage barriers, could indeed increase falling awareness of seniors and provide useful skills to adopt precaution/self-management approaches, and reduce the related risk [13].

Falls in particular have often caused fractures (often femur) and generated some fears in our respondents, such as fear of falling in general/again. Several authors highlight falls as crucial cause of hip fractures [19,20]. Regarding the fear of falling, data from Epicentro [30] reveal that approximately 4 out of 10 seniors are afraid of falling, in particular 7 out of 10 of those who have already fallen. In addition, from our findings also the need for walking aids and to hire a “monitoring” PCA emerged. Regarding this last aspect, previous literature highlights that private/personal assistants are usually hired when seniors have health problems, and after falling with consequent fractures, thus requiring monitoring [74]. As for assistive walking device, some authors report that these can contribute in preventing and decreasing fall incidents, even though the latter can lead to severe injuries when seniors use for instance a wheeled walker [75]. It should however be mentioned that older people who use walking aids were reported to be more affected by fear of falling than older people who do not use them, and this could be linked to their ‘lost confidence in overcoming challenging situations and obstacles in their environment, and this may lead them to reduce to maintain balance or control of gait’ [23] (p. 9). Other authors found that most falls occur while walking [73], and that the use of walking aids is among risk factors for falls [51]. Thus, educating and training seniors in the appropriate use of assistive mobility devices is considered effective in fall prevention [21,75]. Further studies confirm the overall greater fear of falling for community-dwelling older people already experiencing falls [76], and indicate past falls in later life as important predictors of future falls [77].

It is worthy to mention also the feeling of loneliness that, although only in one case in our study, seems to affect mobility after a fall. Living alone seems indeed negatively impact the desire to try anyway to walk, whereas living with a spouse could generate a mutual help in this respect. Accordingly, literature highlights how seniors could risk falling also when they miss the support of a spouse and have symptoms of depression [78]. In particular, some authors [79] indicate that being widower can counteract the willingness to go out in company, to walk without someone who supports physically and morally, including the desire to take care of own health. Widowhood, in later life, can be thus accompanied by psychosocial consequences, e.g., social isolation and depression, both leading to serious health consequences such as chronic medical conditions [80].

### 4.2. Using Communication Technologies for Seeking Help

Overall, our respondents used the mobile phone to communicate, and less the smartphone, both generally and in case of health emergencies. Only three respondents reported the use of remote alarms/devices, and someone expressed some doubt about their wearability. The mobile was particularly useful to call immediately for help in case of falls at home, especially if these occurred while seniors were alone. Some older people remained indeed on the ground for many hours before being rescued, in case they had not the possibility to reach and use a telephone.

Frail seniors living alone, similar to the participants in our study, have thus higher risk for falling and not being found/saved in a short time. In this respect, especially alarm systems could represent a support for home care, by providing an opportunity to contact an emergency center or family members by pressing a button. However, as also emerged from our findings, many authors put in evidence that often older people do not use such alarms to call for help after a fall [81,82], since they consider these devices really intrusive, especially at night, and do not remember to wear them, even though alarms can increase their sense of safety at home [82,83,84]. Acceptance of technologies, aimed at supporting older people living alone, plays a fundamental role for ageing in place [85], and results from a study [38] showed indeed the need of wearable and easy to use alarms, as well as the need to introduce measures to promote the acceptance itself by seniors. Sarlo and colleagues [86] report different results, indicating that technology is almost appreciated among seniors living in North Italy (Bolzano), where the use of personal alarms has been successfully implemented, and whose signals (measurements of parameters and medical alarms) can be sent via text messages or e-mail, by mobile phone, both to family members and a Social Services Company. The latter is responsible for implementing the intervention protocols established for emergencies.

Overall, literature greatly highlights how technology could in principle help frail older people living alone to better cope with emergencies, social isolation and loneliness [87], with great potential to allow independent living/ageing at home, especially of seniors with multimorbidity and living in “difficult to reach” rural sites [88,89]. Moreover, the recent COVID-19 pandemic highlighted the benefits deriving from socialization and interaction of seniors thanks to technology, telephone calls and video calls, in contrast to the physical/social distancing imposed by the lockdown and overall containment measures for tackling the spread of the virus put in place by governments [90]. However, low knowledge of technology and scarce digital skills of seniors, seem crucial [86,91]. In this regard, it is indicative that some of our respondents consider overall mobiles/smartphones even harmful to health, and also they do not exactly know their operating mode and how to use them. According to ISTAT [92], older people in Italy are indeed characterized by a “digital primitivism” profiling them as “basic” users, and only 34% of families composed exclusively of people over 65 years have a broadband connection (vs. 95% of families with at least a child under 18 years). It should also be noted how technology could have an inverse negative effect, in terms of increasing isolation of seniors, as a consequence of fewer face-to-face contacts. In some studies, the prevalence of social isolation was indeed higher when assisted-living arrangements were provided (e.g., telecare) [93,94].

### 4.3. Who Provided the First Help

When health emergencies occurred for our respondents, mainly the parental network intervened (e.g., children and nieces/nephews), but the sudden event was sometimes “managed” also thanks to the first/immediate support of neighborhood/friends. In particular neighbors then proceeded to alert family members or GP after the incident. These results are greatly supported by previous literature. According to Ranci and colleagues [95], family members provide 86% of the help needed by seniors living alone, especially those aged 75 years and over, even though an important support is the neighborhood, which often corresponds to relationships and social contacts consolidated over time. Other authors report that friends and neighbors provide frequent help to seniors, and often have a crucial role also in coordinating other supports [96]. In particular, social exchange with neighbors is facilitated by the proximity of the place of residence, and reduces isolation, especially in later life [97]. In our study also emerged (in three cases) the importance of having provided a copy of one’s home key to relatives and friends/neighbors. Social connection is indeed important for older people, and in this respect, besides family and friends, aging-friendly neighborhoods seem effective, also for promoting their psychosocial well-being [98].

Other persons were further helpful for our respondents, such as passersby/unknown people in case of falls outside the home/on the street. Similarly, in a study from Nyman and colleagues [99] involving 44 seniors aged 65–92 years, 88 episodes of outdoor falls when crossing a road were reported, and in these circumstances old sufferers get help mainly from passersby. It is also to highlight that older adults seem feeling much more confident when walking if they believe they can have support from someone (also unknown) in case they fall [76,100].

The “sad” statement “do not disturb” the family/others, in case of health emergencies, further emerged from our results. In particular, seniors sometimes do not want to bore their children who work, especially when they live far away and could arrive “too late”. Thus, a pro-active capacity to deal independently with an emergency is reported, e.g., to seek promptly for public health help (e.g., GP, emergency room) by themselves. Differently, some interviewees decided to not seek for any type of help when health emergencies were experienced. These extreme solutions seem even a necessity when seniors believe/perceive that family/children cannot be of immediate help, and “do it yourself” represents the effective possibility allowing “not disturbing” anyone. In this respect, some authors specify that, in many cases, older people do not ask for help because they are convinced to have no need in this respect [95], especially seniors with chronic diseases, such as diabetes and heart disease, often representing self-managed illnesses [20,101].

Conversely, two seniors in our study reported to be greatly afraid and worried in case they have to manage a possible night emergency, because in such circumstance they had no one to call for help. Literature highlights the importance of family/social ties for health in old age, and how social interaction is negatively linked to the risk of falling, without of course underestimating the obstacles to mobility related to physical environment [30], although the home is often not perceived by older people as a “dangerous” place in itself [72]. Adequate social support seems therefore fundamental for contrasting the isolation of seniors, and in turn possible risk/consequences of health emergencies, especially falls.

### 4.4. Limitations and Trustworthiness of the Study

The study presents some limitations to be considered. Firstly, it is to premise that, even though the study has been carried out only in three Italian regions, they are however representative of different socio-economic development levels of this country. Thus, the fact that findings cannot represent the whole Italian context, could be partially considered as a limitation. Additionally, since this study provides an overall picture of the health emergencies and falls experienced by older people, in the analysis a comparison between the different regions is missing, thus possible higher falls rate reported in a region, or geographical discrepancies in technology utilization in this respect, are not addressed. This means lacking useful information preventing from designing interventions tailored for specific regions. Secondly, the definition of frailty is not exhaustive but limited to old age (65 years and over), ageing alone in place, and presence of functional limitations, leading seniors to need support for the activities of daily living [102]. Thus, no standard tool was used to assess frailty, and this dimension was ‘asked’ instead of measured. Thirdly, the cognitive assessment of interviewees was based only with the information from the recruitment channels, then confirmed by the respective families. Fourthly, the study considers health emergencies that occurred in the last three years (before the interview) and as such presents biases and pitfalls linked to retrospective survey questions, since respondents might have forgotten some details. Moreover, they tend to answer less accurately when asked about their past, thus reporting feelings that might be more linked to their current life [103]. Fifthly, an analysis of the use of communication technology to ask for help in case of health emergency, and of relatives/neighbors who gave help, in relation to different circumstances (e.g., various illnesses and falls) is lacking, since responses were often recorded as a whole in this respect. Sixthly, since the sample is composed by more women than men (90 vs. 30), the gender dimension was not explored, even though this could overall provide further insights, as supported by some studies showing how older men are less likely to fall than older women [104,105]. In addition, the gender-associated technology-affinity could be explored, that is, as stated by some literature, men seem having a more natural affinity with technology then women [106], and this could be reflected in a higher willingness to adopt technical aids for preventing falls. In addition, since our study focused on the Italian context, we have reported/discussed some non-English papers/some Italian national statistics (e.g., from ISTAT, Italian National Institute of Statistics), for a comparison with our findings, when relevant. This however limits the readability from a wide international public. Finally, absolute values in tables should be interpreted with caution, since they are sometimes very low.

Despite these limitations, credibility, transferability, dependability and confirmability of our study, as overall trustworthiness of the qualitative analysis, is supported, as indicated by Lincoln and Guba [107]. The credibility regards the use of a topic guide partly based on questionnaires successfully applied in previous studies on older people (e.g., with functional limitations and need of support for performing ADLs and IADLs) [47], and on peer de-briefing sessions among researchers (in order to define protocol/topic guide, data collection-analysis, and discuss findings) who have a prolonged experience regarding the issue of ageing in place. Moreover, dissemination seminars with stakeholders and experts allowed to validate preliminary results. The transferability of qualitative analysis [108] is based on a deep preliminary literature review [109] and analysis of several studies on the topic of ageing in place [95], as background data for the initial conceptual/multidimensional framework [34]. Finally, the dependability and confirmability of the research results, i.e., the use of replicable methods, were supported by a detailed description of the study protocol (approved by a Bioethics Committee), including several specifications on data collection and analysis process. For more details on limitations and trustworthiness of the main “IN-AGE” study, further information can be found in a previous publication [33], from which these aspects are partially adapted.

## 5. Conclusions

The frailty conditions of older people, with physical limitations, imply the occurrence of possible emergency situations, which can significantly compromise their health, especially when living alone without cohabiting relatives. Apart from episodes regarding various illnesses suffered, falls are reported very frequently. In such a context, it seems crucial the use of communication technology to seek for available help. However, in case of health emergencies, some older people ask for help, but other do not, also to not disturb the family, especially children. The exploration of these aspects, with regard to Italian seniors, has provided an overall picture of risks and needs of support of an ageing population, and can offer new useful insights for policy makers, for prevention and management of emergencies.

In particular, since our study highlights that falls mainly happen at home, thus representing an urgent public health concern with damaging consequences, community-based interventions should be designed, allowing older people to live independently as much as possible. However, also falls outdoor occurred, and overall, fall prevention strategies should emphasize safer environments/homes, community infrastructure, improved accessibility of walkable public spaces, road crossings allowing to cross safely, balance/gait training, appropriate use of assistive devices, and appropriate policies and legislation [18]. Moreover, public health policies should aim at reducing the fear of falling, that can negatively impact the concrete possibility of ageing in place. In addition, emergencies prevention in general, not only falls, should consider wearable (i.e., they do not reduce mobility) personal alarms, fall sensors and mobile phones with SOS emergency buttons. These assistive devices, particularly alarms, emerged as rarely used by our respondents. In this respect, the overall use of technological tools among seniors should be enhanced, also by means of awareness campaigns and dedicated training programs, in order to improve/increase their acceptance.

Finally, more fall-related research seems in particular needed, in order to inform the design of policies to reduce risk and prevent falls among older people, also by identifying existing effective good practices. Furthermore, the overall construction of more age-friendly housing, cities and communities [110], could benefit from building/increasing awareness of the importance of falls prevention in older people, family caregivers, health professionals, and service providers.

## Figures and Tables

**Table 1:** Overview of macro-categories, sub-categories, and labels.

Macro-Categories	Sub-Categories	Labels
Health emergencies in the last 3 years	Physical complaints/illnesses	Heart/circulatory problem, heart attack Breathing problems, cough, bronchitis Joint problems Stroke/Ischemia Other: flu, tonsillitis, fainting, inflammation, anxiety
	Falls	In the home, outside the home Consequences: fractures, fear of falling, fear of broken sidewalks and streets, need of aids, need to hire a personal care assistant (PCA)
Use of Communication Technologies	General use	Only landline telephone Mobile, Smartphone Remote alarm/call devices: used, not owned/wanted, not owned/not wanted
	To ask for help in case of (reported) emergencies	
First help for health emergencies	From relatives/community	Family: children, nieces/nephews, sisters/brothers Friends/Neighbors PCA Others: passersby, unknown people Copy of the home keys to family/friends/neighbors
	‘Do-it-yourself’	To seek for public health help by themselves contacting emergency room and/or General Practitioner (GP) No help sought Reasons: “do not disturb/worry” the family, family cannot be of help

**Table 2:** Sample Characteristics (absolute values/*n* and %).

Characteristics	N = 120	%
**Age** (years)		
67–79	36	30
80 and over	84	70
**Gender**		
Male	30	25
Female	90	75
**Education ^1^**		
No title	14	11
Primary-Middle school (5 and 3 years)	75	63
High School-University (3–5 years both)	31	26
**Marital Status**		
Single/Divorced ^1^	32	27
Widowed	88	73
**Living Situation**		
Alone	93	78
With PCA	27	22
**Level of physical limitations ^2^**		
Mild/Moderate	63	53
High/Very High	57	47
**Mobility**		
Only/Mainly in the home ^3^	48	40
Also outside the home with help ^4^	72	60
**Care arrangements/Supports ^5^**		
Help from Family *(Children)*: yes	94 *(71)*	78 *(60)*
Help from Public Services (*Home care*): yes	43 *(28)*	36 *(23)*
Total Cases/Respondents	120	100

^1^ This includes two male respondents still married but not cohabiting with their spouses. ^2^ The level of physical/functional limitations is based on 12 ADLs-IADLs, two mobility limitations (going up/down the stairs and bending to pick up an object), plus sensory limitations in hearing and seeing. Mild = no activities “not able”, Moderate = 1–2, High = 3–4, Very high = 5 or more. ^3^ This includes also respondents able to move outside the home very rarely, i.e., less than two times a week and only if accompanied or with aids (cane, walker). ^4^ Respondents are able to move within the home and also outside at least two times a week, only if accompanied or with aids (cane, walker). ^5^ In some cases both supports (family and public services) were reported (the sums of absolute and percentage values are higher, respectively, than 120 and 100).

**Table 3:** Health Emergencies in the last three years (at least one episode).

Emergencies ^1^	n. of Statements
**Physical Complaints/Illnesses ^2^**	50
Heart/circulatory problem, heart attack	20
Breathing problems, cough, bronchitis	11
Joint problems	8
Stroke/Ischemia	6
Other ^3^	5
**Falls**	47
In the home	36
Outside the home ^4^	11
**No episode of health emergency (last 3 years)**	32

^1^ Emergencies were referred by 88 participants, and more episodes in some cases were reported (for a total of 97 episodes). ^2^ These are episodes occurred mainly inside the home. However, not always a distinction in/outside the home is reported by respondents in this respect. ^3^ Flu, tonsillitis, fainting, inflammation, anxiety; ^4^ 7 falls in the street, 2 in hospital, 1 in a shop, 1 in a church.

**Table 4:** Consequences of falls.

Consequences	n. of Statements
Fractures	28
Fear of falling	18
Fear of broken sidewalks/streets	8
Need of aids	6
Need to hire a PCA	3

**Table 5:** Independent use of technologies: in general, and for emergencies ^1^.

Technologies ^2^		n. of Statements
	General use	To ask for help in case of emergencies
Landline telephone only	19	-
Mobile	80	40
Smartphone	21	5
Remote alarm/call devices ^3^	13	3

^1^ Analysis regards 88 participants who referred at least one episode of health emergency. ^2^ More types of technologies were used in some cases; ^3^ 4 respondents have a mobile with SOS option, and 1 used it in case of emergency.

**Table 6:** First help for health emergencies (at least one type of help) ^1^.

Help ^2^	n. of Statements
**Family** ^3^	52
Children	38
Niece/Nephew	9
Sisters/Brothers	5
**Friends/Neighbors**	21
**PCA**	4
**Others** ^4^	14
Passersby/unknown people	8
**‘Do-it-yourself’**	17
To seek for public health help by themselves	11
No help sought	6

^1^ Analysis regards 88 participants who referred at least one episode of health emergency. ^2^ The values in the table do not concern the number of family members, friends, etc. who have been of help in emergencies, but the number of older people who reported at least one help of the respective type in these circumstances. ^3^ More types of help in some cases were reported (e.g., family and friends). ^4^ This includes also 3 social workers/operators, 2 medical staff, and 1 shop clerk.

## Data Availability

The qualitative data supporting the findings of the study and original verbatim transcriptions in the thematic charts are not publicly available due to privacy/ethical restrictions. There is indeed confidential information that could compromise the anonymity of research participants as potential identifiers of respondents, e.g., names of persons and city of residence. However, a dataset regarding the sample of the main study carried out in 2019 is openly available in Mendeley at https://doi.org/10.17632/3ryrpz224h.2 (accessed on 26 July 2022).
